# Alzheimer's disease plasma biomarkers in a global cohort: experience from the DAWN study

**DOI:** 10.1002/alz70856_105522

**Published:** 2026-01-08

**Authors:** Anthony J Griswold, Rufus O. Akinyemi, Farid Rajabli, Biniyam A. Ayele, Motunrayo Coker, Kyle M Scott, Kazeem Akinwande, Larry D Adams, Samuel Diala, Patrice G Whitehead, Jacob L. McCauley, Mayowa Ogunronbi, Kara L Hamilton‐Nelson, Albertino Damasceno, Andrew F Zaman, Yared Z Zewde, Alfred Kongnyu NJAMNSHI, Allison M Caban‐Holt, David Ndetei, Fred Stephen Sarfo, Joshua O Akinyemi, Susan H. Blanton, Albert Akpalu, Michael L Cuccaro, Kolawole Wahab, Seid Ali Gugssa, Katalina F. McInerney, Reginald Obiako, Olusegun Baiyewu, Richard Walker, Pedro R Mena, Njideka U Okubadejo, Izri M Martinez, Brian W Kunkle, Stella‐Maria Paddick, Raj Kalaria, Adesola Ogunniyi, Jeffery M Vance, Christiane Reitz, Sudha Seshadri, Maëlenn Guerchet, Giuseppe Tosto, Scott M Williams, William S Bush, Jonathan L Haines, Goldie S Byrd, Margaret Pericak‐Vance

**Affiliations:** ^1^ John P. Hussman Institute for Human Genomics, University of Miami Miller School of Medicine, Miami, FL, USA; ^2^ Dr. John T. Macdonald Foundation Department of Human Genetics, University of Miami Miller School of Medicine, Miami, FL, USA; ^3^ College of Medicine, University of Ibadan, Ibadan, Oyo State, Nigeria; ^4^ Institute of Advanced Medical Research and Training, College of Medicine, University of Ibadan, Ibadan, Oyo State, Nigeria; ^5^ John P. Hussman Institute for Human Genomics, University of Miami Miller School of Medicine, Miami, FL, USA, Miami, FL, USA; ^6^ Addis Ababa University, Addis Ababa, Addis Ababa, Ethiopia; ^7^ Global Brain Health Institute, San Francisco, CA, USA; ^8^ College of Medicine, University of Ibadan, Ibadan, Oyo, Nigeria; ^9^ University of Miami Miller School of Medicine, Miami, FL, USA; ^10^ Dr. John T. Macdonald Foundation Department of Human Genetics, University of Miami, Miami, FL, USA; ^11^ John P. Hussman Institute for Human Genomics, University of Miami, Miami, FL, USA; ^12^ Faculty of Medicine, Eduardo Mondlane University, Maputo, Mozambique; ^13^ College of Health Sciences, Addis Ababa University, Addis Ababa, Ethiopia; ^14^ Brain Research Africa Initiative, Yaounde, Centre, Cameroon; ^15^ The University of Yaoundé I, Yaoundé, Cameroon, Cameroon; ^16^ Wake Forest University School of Medicine, Winston‐Salem, NC, USA; ^17^ University of Nairobi, Nairobi, Kenya; ^18^ Kwame Nkrumah University of Science and Technology, Kumasi, Ghana, Kumasi, Ghana; ^19^ University of Ghana Medical School, Accra, Ghana; ^20^ Division of Neurology, Department of Medicine, Ilorin, Nigeria; ^21^ Addis Ababa University, Addis Ababa, Ethiopia; ^22^ Department of Neurology, University of Miami Miller School of Medicine, Miami, FL, USA; ^23^ Ahmadu Bello University, Zaria, Kaduna, Nigeria; ^24^ College of Medicine, University of Ibadan, Ibadan, Nigeria; ^25^ Translational and Clinical Research Institute, Newcastle University, Newcastle, Newcastle Upon Tyne, United Kingdom; ^26^ College of Medicine, University of Lagos, Lagos, Nigeria; ^27^ Institute of Neuroscience, Newcastle University, Newcastle, Newcastle Upon Tyne, United Kingdom; ^28^ Gateshead Health NHS foundation Trust, Department of Old Age Psychiatry, Gateshead, Newcastle Upon Tyne, United Kingdom; ^29^ Translational and Clinical Medicine, Newcastle University, Newcastle upon Tyne, United Kingdom; ^30^ Newcastle University, Translational and Clinical Research Institutetitute for Ageing and Health, Newcastle UniversityNewcastle University, Newcastle upon Tyne, United Kingdom; ^31^ Gertrude H. Sergievsky Center, Taub Institute for Research on the Aging Brain, Departments of Neurology, Psychiatry, and Epidemiology, College of Physicians and Surgeons, Columbia University, New York, NY, USA; ^32^ Glenn Biggs Institute for Alzheimer's & Neurodegenerative Diseases, University of Texas Health Science Center, San Antonio, TX, USA; ^33^ LEMACEN, Faculty of Health Sciences, University of Abomey‐Calavi, Cotonou, Other, Benin; ^34^ Case Western Reserve University School of Medicine, Cleveland, OH, USA; ^35^ Department of Population and Quantitative Health Sciences, Institute for Computational Biology, Case Western Reserve University, Cleveland, OH, USA; ^36^ Cleveland Institute for Computational Biology, Department of Population & Quantitative Health Sciences, School of Medicine, Case Western Reserve University, Cleveland, OH, USA

## Abstract

**Background:**

The DAWN study aims to investigate Alzheimer's disease (AD) genetics and underlying biological markers in a global population including African Americans (AA: 4,000) and Hispanic/Latinos (HI: 4,000) ascertained in the US and indigenous Africans (AF: 5,000) through collaboration with the African Dementia Consortium (AfDC) from 10 African counties. These 13,000 participants include AD cases and individuals with mild (MCI) or no cognitive impairment (NCI). To understand the underlying blood‐based AD biomarkers profile of this unique cohort, we are analyzing the plasma levels of pTau181, neurofilament light chain (NFL), and Glial fibrillary acidic protein (GFAP).

**Method:**

We measured pTau181 and NFL, and GFAP with Simoa chemistry using the pTau181 AdvantageV2 and NEUROLOGY 4‐PLEX A assays, respectively, on the Quanterix HD‐X instrument. Our preliminary cohort consisted of 174 AF (86 AD; 88 NCI) from Nigeria and Ghana study sites, 254 AA (24 AD; 102 MCI; 85 NCI), and 166 HI (44 AD; 61 MCI; 62 NCI). Linear mixed‐effect regression models adjusted for age, sex, population substructure and relatedness followed by Bonferroni correction were applied to identify biomarker differences.

**Result:**

There were no significant differences between the ancestral groups within the diagnostic categories for any of the biomarkers measured. Plasma pTau181 concentrations were increased in AD relative to NCI in all three populations (*p* = 5.1x10^‐4^, 9.6x10^‐5^, 5.1x10‐4 in AA, AF, and HI respectively) and AD relative to MCI (*p* = 0.012, 0.0014 in AA and HI respectively), though no differences were noted between MCI and NCI. Interestingly, GFAP and NFL were highly significantly increased in AF AD vs NCI (*p* = 4.5x10^‐9^, 7.9x10^‐7^ for GFAP and NFL respectively) and in HI (*p* = 7.9x10^‐8^, 1.6x10^‐6^ for GFAP and NFL respectively), but no differences noted in AA.

**Conclusion:**

These results suggest AD biomarkers are generalizable across global populations, with baseline values being consistent. However, there are notable differences, particularly in NFL and GFAP levels, which may reflect underlying differences in environmental or genetic influences on AD. Ultimately, increasing sample sizes and combining genomic, biomarker, and social and environmental data will increase understanding of genetic risk of AD.

